# Machine learning-assisted paper-based chemiluminescence biosensor for choline quantification in infant milk: toward portable nutritional quality monitoring

**DOI:** 10.1038/s41598-026-50484-4

**Published:** 2026-04-29

**Authors:** Jitendra B. Zalke, Vani Kaushik, Chirag M. Singhal, Madhusudan B. Kulkarni, Manish Bhaiyya

**Affiliations:** 1 Department of Electronics Engineering, Ramdeobaba University, Nagpur, Maharashtra 440013 India; 2 Manipal Institute of Technology, Manipal Academy of Higher Education, Manipal, India; 3 Department of Electronics and Telecommunication, Shri Sant Gajanan Maharaj College of Engineering, Shegaon, 444203 India

**Keywords:** Paper-based senosrs, Chemiluminescence, Biosensor, Choline detection, Infant milk analysis, Machine learning, Smartphone-integrated sensing, Point-of-care testing, Nutritional quality monitoring, Biochemistry, Biological techniques, Biomarkers, Biotechnology, Neuroscience

## Abstract

Choline is a critical micronutrient that is involved in neurotransmission, lipid metabolism, and neurodevelopment of infants. Poor consumption of choline in the initial stages of growth might lead to severe cognitive and developmental defects. Hence, there is a need to constantly monitor choline level in infant food. To meet this requirement, we present a low-cost paper-based luminol cobalt chemiluminescense (PLC-CL) biosensor with the potential to analyze choline in milk and in real-time using smartphone imaging and machine learning (ML) algorithms. The sensing platform works for the detection of the hydrogen peroxide (H_2_O_2_) which is generated through the use of choline oxidase (ChOx), and then the produce a luminol-cobalt chemiluminescent reaction, where the intensity of the emitted light is directly proportional to the concentration of choline. ML models quantify light intensity, and are highly accurate without the need to use standard methods of calibration. This device has a linear dynamic range of 0.5–10 mM and a detection limit of 257.12 µM, thus it is useful in quantification in real time and can also be used in the real field envirnoment. This point-of-care testing (PoCT) biosensing tool, enriched with an ML algorithm, therefore, increases nutritional surveillance functions, especially in resource-limited environments, and contributes to adherence to the infant food safety protocols..

## Introduction

The safety and quality of infant food preparations in terms of nutritional value have emerged as a popular public health issue in the beginning of the twenty-first century. Choline is a vital micro nutrient, which plays a role in brain development, neurotransmitter, lipid metabolism, and cell-membrane integrity^1–5^. It is a biochemical precursor to acetylcholine-an essential neurotransmitter involved in memory and thinking. Also, it is involved in the formation of phosphatidylcholine and sphingomyelin which are important parts of neuronal membranes. The lack of choline in the early development of a child can develop into neurodevelopmental disorders, liver dysfunction, and cognitive immaturation, which is why it is imperative to regularly check infant milk and formula feeds. Human milk has reported total choline concentrations that are estimated to be 125–166 mg/L, or 1.2–1.6 mmol/L total choline. Infant formulas also include choline, measured at a wide range of about 82–209 mg/L, depending on formulation and brand. These values indicate that physiologically relevant choline concentrations in milk and formula lie in the high micro-molar to low milli-molar range^1,6–9^. Hence, it is important to have reliable and additional, quantifiable choline to comply with regulations and infant health^10–12^.

Traditionally, choline has been detected using complex laboratory techniques, such as gas chromatography-mass spectrometry (GC–MS), liquid chromatography-tandem mass spectrometry (LC–MS/MS), and high-performance liquid chromatography (HPLC). Despite being highly accurate and sensitive, these methodologies suffer from several drawbacks, including being expensive, requiring a considerable amount of time, necessitating a competent operator, and involving tedious sample preparation. They require large instrumentation and are not applicable to on-site or real-time testing due to the need for heavy machinery and the requirement to maintain a controlled laboratory setting, which is often not feasible in small-scale food production or rural manufacturing environments where cost and simplicity are the primary considerations. Such shortcomings demonstrate the growing necessity of a handheld, inexpensive, and easy to use alternative of analysis with the ability to find the concentration of choline in a relatively short amount of time with comparable accuracy^6,13–19^. The chemiluminescence based biosensing platforms have generated much attention in the last few years, due to their intrinsic features such as its high sensitivity, low background noise, and a large dynamic range. Compared to colorimetric or fluorescence detection, chemiluminescence detection results in the production of light only by the means of a chemical reaction and does not need external sources of excitation, which helps to reduce signal interference^20–24^.

The chemiluminescent choline assays that are most commonly used are based on the principle of choline oxidase (ChOx) catalyzing the oxidation of choline, by which hydrogen peroxide (H_2_O_2_) is produced as a by-product. This produced H_2_O_2_ is reacted with luminol in the presence of transition-metal catalysts (e.g., Co^2+^) to produce light emission which can easily be detected. The emitted luminescence intensity is directly proportional to the choline concentration and therefore it can be quantified selectively and sensitively. However, the applications in practice usually use the chemiluminescence devices that are photomultipliers or CCD-driven which are expensive, power-gathering and cannot be utilized in the field^25–32^. The potential approach to eliminate these limitations is the introduction of chemiluminescence chemistry to paper-based analytical devices (PADs). Paper substrates are good in density, biodegradability low cost and support passive microfluidic flow through capillary action, so no pumps or external power sources are required. Together with miniature and handheld passive optical imaging devices like smartphone cameras, paper-based chemiluminescent sensors offer a promising future of cheap, disposable point-of-care testing (PoCT)^33–36^.

Despite these advances in chemiluminescence detection using a smartphone, there is still a source of variation, which is due to the difference in lighting, camera parameters, as well as background noise. Such inconsistencies have an impact on accuracy and repeatability of the analytical procedure. Development of machine learning (ML) has offered a powerful computational method of biosensing that has overcome these challenges. ML algorithms have the capability to condition on complicated nonlinear images, normalize to environmental variation and predict analyte concentrations with maximum accuracy. The ML component serves as a data-driven analytical tool that enhances the practicality and usability of the biosensing platform, rather than proposing a new algorithm. ML models, especially regression-based algorithms, including but not limited to: polynomial regression, gradient boosting, and support-vector regression (SVR), allow quantifying robustly and repeatable without manual calibration, using large arrays of chemiluminescent images. This type of integration gives the traditional sensors the ability to integrate with smart analytic systems that can interpret data on demand and automatically^23,37–41^.

The proposed research provides a new PLC-CL biosensor with smartphone imaging and ML features that can be used to quickly and locally quantify choline in infant milk. The proposed system incorporates three components that are synergistic, namely, a luminol-cobalt-based CL chemistry system to generate signals, the use of a smartphone camera to provide portability in imaging, and ML algorithms to enable automated quantification. The intelligent sensing architecture ensures the accuracy, portability, and cost-effectiveness of analytics, in addition to meeting the essential requirement of AI-supported, point-of-care biosensors for nutritional and food safety testing. More than analytical power, however, the PLC-CL platform embodies a move towards democratised diagnostics, as it enables industry and public health authorities to perform real-time nutrient measurements without advanced laboratory facilities.

## Materials and methods

**Fig. 1:**
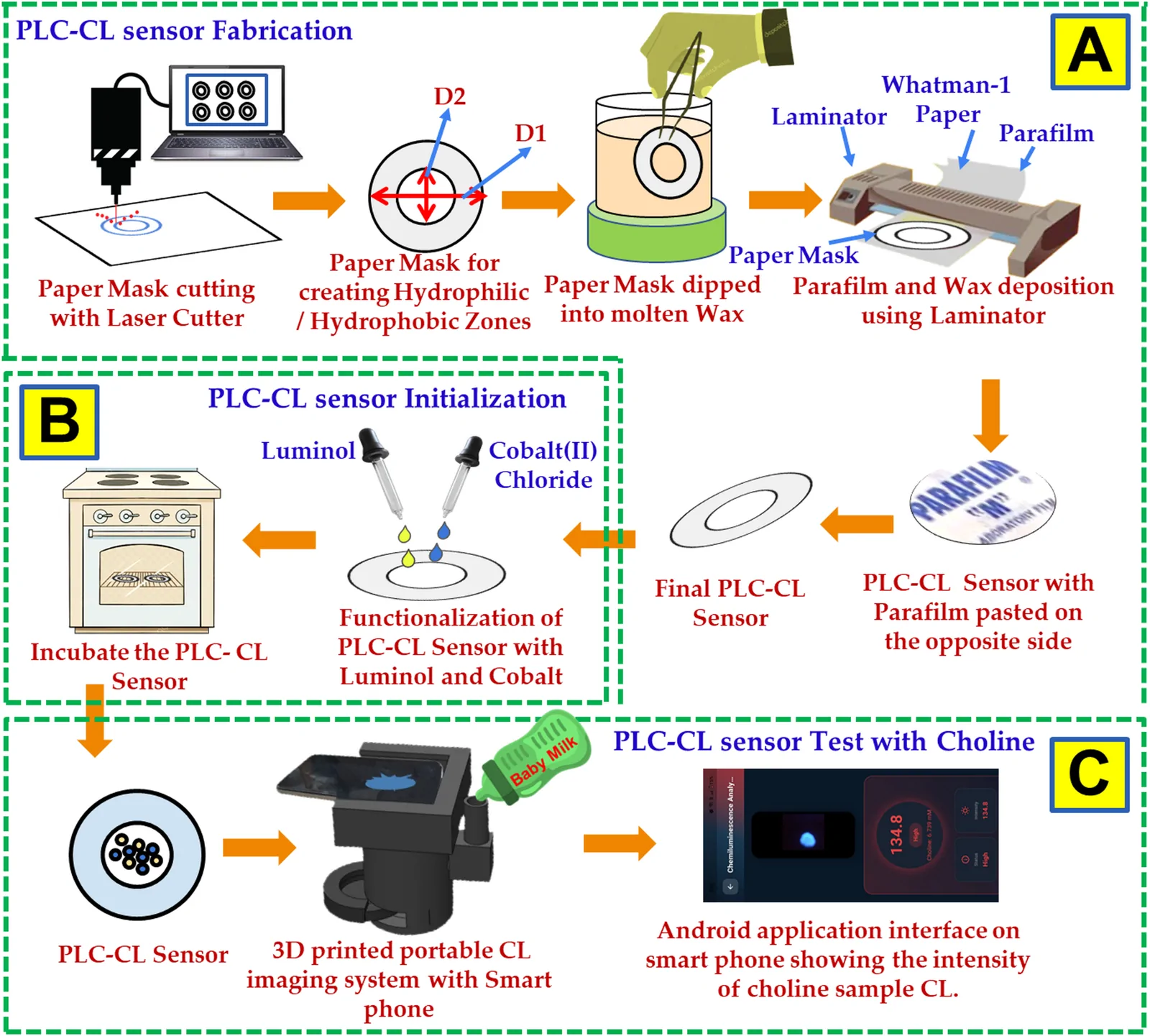
Illustrated workflow of a machine learning-supported portable chemiluminescence biosensor, detailing the sequence from choline sensing in food samples (milk) to analytical processing on a 3D-printed platform integrated with a smartphone.

This section describes various chemicals used, Fabrication of the PLC-CL sensor, PLC-CL sensor functionalization, working mechanism, and the AI-assisted CL imaging and quantification framework in the proposed work, as illustrated in Fig. 1.

### Chemical materials used

Choline, Choline Oxidase (ChOx), Luminol (C_8_H_7_N_3_O_2_) ≥ 97% (HPLC grade), Cobalt (II) chloride (CoCL_2_), Hydrogen Peroxide (H_2_O_2_), Sodium Carbonate (Na_2_CO_3_), sodium bicarbonate (NaHCO_3_) ≥ 99.7%, Lithium Lactate, Lactose, Glucose, calcium, phosphorus, Starch, Iodine, deionized water (DI), Whatman grade-1 paper, A KYLIE Pro Wax100 warmer hot wax heater, a VMS professional LM deluxe heavy-duty lamination machine, and a hot and cold A3 laminator were used.

### Fabrication of paper-based luminol-cobalt chemiluminescence (PLC-CL) sensor

Figure 1, provides a step-by-step illustration of the whole fabrication workflow. The Whatman Grade-1 filter paper was selected as the primary substrate for the development of the PLC-CL biosensor for choline detection because of its exceptional porosity and fluid-handling characteristics. Luminol and Cobalt(II) chloride (CoCl_2_) were used as chemiluminescent reagents on the biosensor pad, creating a PLC-CL sensor. Hydrophilic zones, where the reagents reacted, and hydrophobic barriers that controlled the fluids were defined using the wax printing technique. This technique is highly commendable due to its ease of handling, low production cost, and scalability in creating biosensors, from laboratory prototypes to field-ready tools. Production processes were carefully designed to ensure accuracy and consistency. The choice pattern was cut out at the beginning of the project using a laser cutter. The mask was accurately placed over circular filter paper pads measuring 20 mm in diameter (D1), whereas the sensing area diameter, which is the internal circle diameter, is 15 mm (D2), as illustrated in Fig. 1A. To form a strong barrier of wax, the A4 sheets of paper were cut into perfect shapes by a laser cutter and then dipped in molten wax to provide the hydrophobic aspect. By placing the wax-patterned paper over the designated conductivity or reaction zones on the filter paper pad, an effective boundary was created to avoid cross-contamination and undesirable lateral fluid movement.

After this was done, the complete assembly was put through a hot laminator. The filter paper was effectively and permanently sealed off from the hydrophilic reaction area by the wax, which had melted to a point where it could pass through. Finishing the biosensor required covering it with a thin layer of parafilm for protection, which increased its mechanical strength and longevity. A dependable CL biosensor made of paper that can detect choline with high sensitivity was the end product of this methodical process. Simple fabrication processes demonstrated the viability of creating disposable, low-cost diagnostic devices well-suited for point-of-care applications, and the distinct separation of hydrophilic and hydrophobic regions with improved fluid management.

### PLC-CL sensor functionalization and working mechanism

**Fig. 2:**
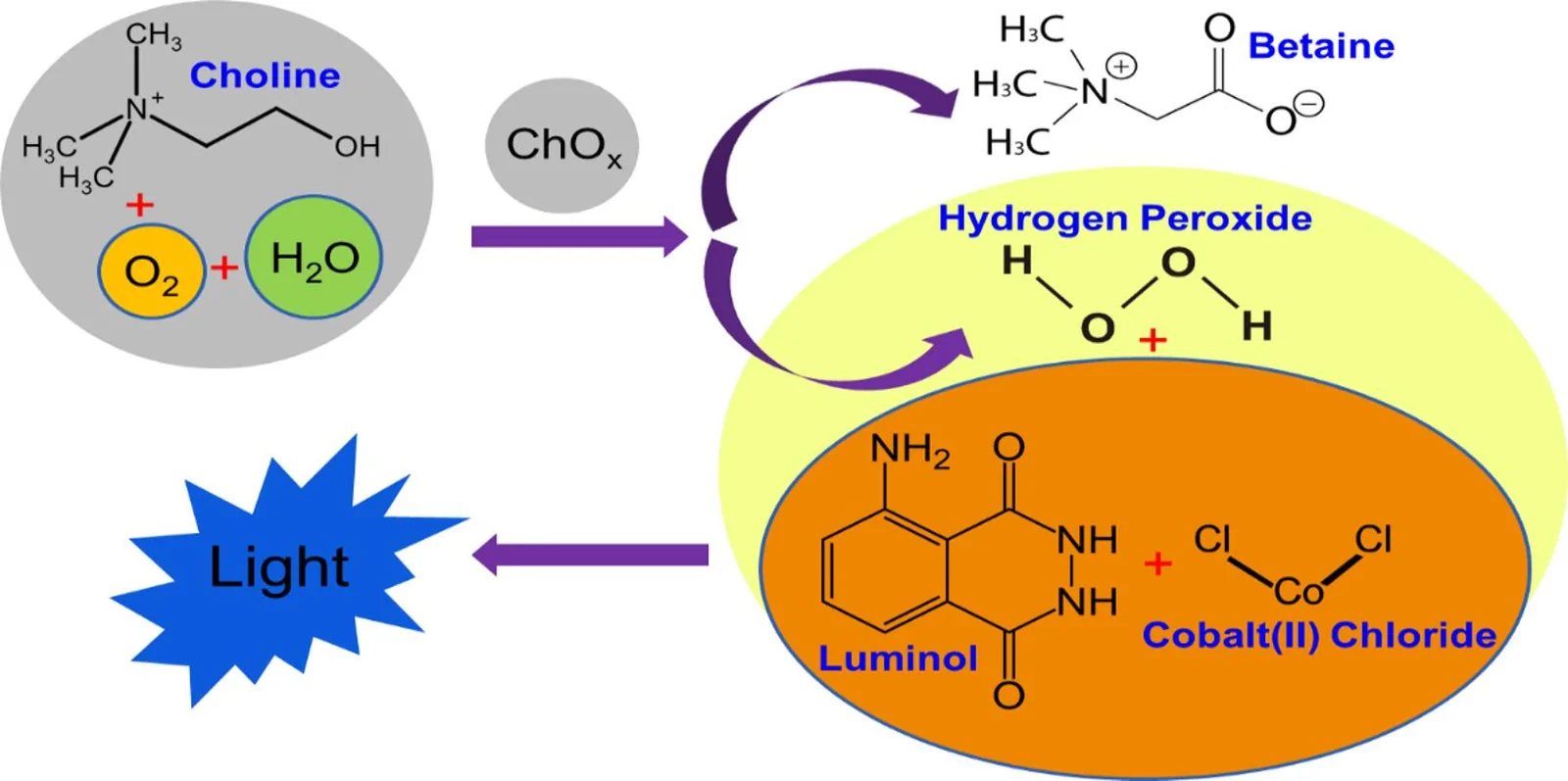
The detailed test procedure and working principle of the PLC-CL sensor chemiluminescence biosensor are fully described below and visually represented in the schematic diagram.

The PLC-CL biosensor was functionalized with various chemical regents after its fabrication. A 3 mM optimised luminol and 3 mM Cobalt(II) chloride concentration was used to functionalize the fabricated PLC-CL biosensor as shown in Fig. 1B. After this, the functionalized device was placed in an incubator set at 70 °C for 25 min to ensure it was activated correctly. As shown in Fig. 1C, the manufactured sensor was put inside a 3D-printed dark enclosure for the purposes of measurement and analysis. The liquids were able to mix and react by staying contained inside a circular sensing region, which was made possible by hydrophobic wax barriers. Because of this, the chemiluminescent process could begin, as the enzyme oxidised choline to H_2_O_2_, as illustrated in Fig. 2.

This system employs choline oxidase (ChOx) and choline to produce reactive oxygen species (ROS) for the luminol chemiluminescent reaction, instead of external hydrogen peroxide (H_2_O_2_), in the luminol/Cobalt (II) chloride (CoCl_2_)/ChOx/Choline-based CL process. This biosensor relies on ChOx to convert the analyte choline into the by-products betaine and H_2_O_2_^42^. Given that choline is not intrinsically chemiluminescent, this enzymatic step is critical. However, as shown in Eq. (1), the luminol-based CL system relies on its oxidation product, H_2_O_2_, as a critical reactant. This means every choline molecule generates a stoichiometric amount of hydrogen peroxide, providing a quantitative link between choline concentration and H_2_O_2_ production as shown in Eq. (1):

1 $$Choline + O_{2} + H_{2} O{\mathop{\longrightarrow}\limits^{{ChO_{x} }}} Betaine + H_{2} O_{2}$$

Luminol acts as the primary chemiluminescent reagent, emitting visible blue light upon oxidation in an alkaline medium^43^. In this process, luminol deprotonates to form a reactive dianion, which is oxidized by H_2_O_2_—generated from ChOx—in the presence of cobalt ions, producing excited 3-aminophthalate that emits light upon relaxation^43^.

2 $$Luminol^{2 - } + H_{2} O_{2} {\mathop{\longrightarrow}\limits^{Catalyst}} 3 - Aminophthalate^{*} + Light \left( {h\gamma } \right)$$

Cobalt(II) ions are used as a catalyst to enhance the reaction between luminol and H_2_O_2_. Transition metal ions, including Co^2+^, are ideal catalysts of the breaking up of H_2_O_2_ into reactive oxygen species, e.g., hydroxyl radicals (•OH) and superoxide anions (O_2_•⁻). Reactive oxygen species are rapid oxidisers of luminol, thereby accelerating the reaction rate and increasing the intensity of light^44^. When no catalyst is present, the rate of the luminol-H_2_O_2_ reaction is slow, and a lower level of luminescence is obtained. Therefore, the application of CoCl_2_ increases both the sensitivity and signal-to-noise ratio of the biosensor. The intensity of the emitted light is directly proportional to the choline concentration and can be readily measured by a smartphone camera for further analysis.

### AI-assisted chemiluminescence imaging and quantification framework

**Fig. 3:**
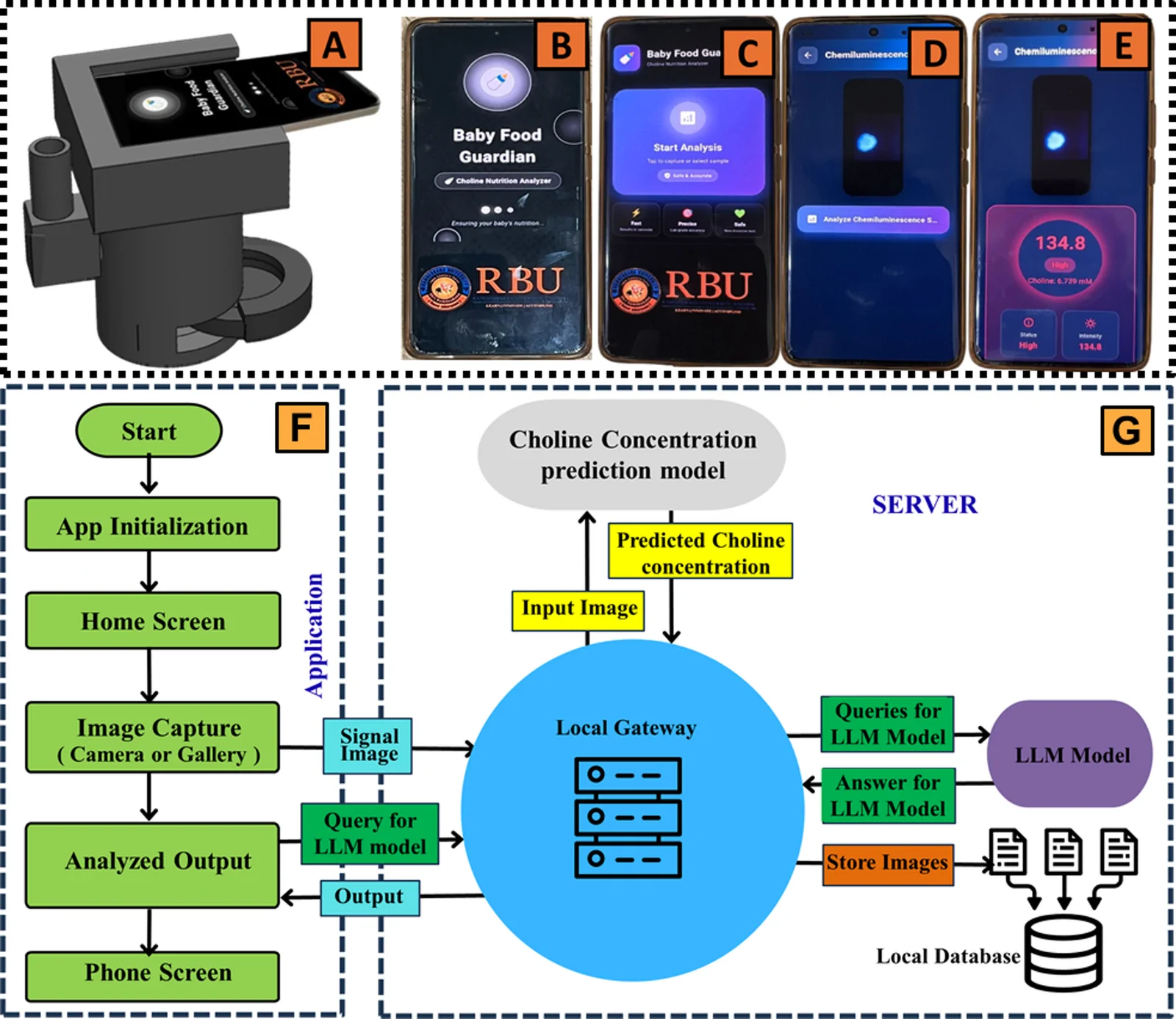
Schematic representation and demonstration of the developed 3D-printed portable chemiluminescence (CL) imaging system integrated with an Android-based application. (**A**) illustrates the 3D-printed portable CL setup, (**B**–**E**) display the graphical user interface (GUI) of the custom-developed Android application for system control and data acquisition, and (**F**) presents the algorithmic workflow implemented for image processing and analysis. (**G**) illustrated the processing at server side for choline concentration prediction mode.

Figure 3 illustrates the complete CL imaging and analysis platform, consisting of the 3D-printed portable sensing chamber integrated with a smartphone for chemiluminescence capture and analysis. The experimental work was carried out using a Moto G35 5G smartphone equipped with a 50 MP + 8 MP rear camera and powered by the UNISOC T760 processor, which provided sufficient computational capability for real-time execution. The entire application was designed in Flutter, allowing seamless integration of image acquisition, processing, and prediction modules within a single mobile environment. For data collection, samples with concentrations ranging from 0.1 to 20 mM were introduced into the setup, and the emitted CL signal was repeatedly recorded using the app through the Flutter camera package to ensure dataset reliability and repeatability.

To prepare the captured images for analysis, a structured preprocessing pipeline was applied to every sample image. Since chemiluminescent intensity is primarily represented through brightness rather than color information, the acquired RGB images were first transformed into grayscale format. This conversion simplified the three-channel image into a single luminance channel directly related to emitted light intensity, thereby reducing computational overhead and improving feature consistency. Within the Flutter framework, this operation was achieved using the image package through the ***img.grayscale()*** function, which efficiently converted each frame into an intensity-based representation suitable for subsequent analysis. Following grayscale conversion, the relevant sensing portion of the image was isolated by defining a fixed Region of Interest (ROI) corresponding to the active paper-based reaction zone. Restricting the analysis only to this chemically responsive region prevented unnecessary background pixels from influencing the extracted measurements. It also ensured that all images were processed under identical spatial conditions. The ROI extraction stage was implemented using the ***img.copyCrop()*** method from the Flutter image library, enabling precise cropping of the same area from every captured image.

Once the reaction zone was isolated, background correction was performed to eliminate unwanted contributions from ambient illumination, sensor offsets, and surrounding dark regions. A baseline intensity value was estimated from non-reactive pixels and then subtracted from the ROI signal so that only the true chemiluminescent response remained. In the Flutter application, this step was executed through pixel-wise manipulation using ***img.getPixel()*** to read intensity values and ***img.setPixel()*** to write corrected values after subtraction. Such direct pixel access made the correction process accurate and computationally lightweight. After baseline removal, intensity normalization was introduced to compensate for slight variations in exposure, camera sensitivity, or minor illumination differences between repeated captures. This step ensured that the intensity values obtained from different images could be compared on a common scale. The normalization logic was implemented using Dart-based arithmetic operations, where each pixel or extracted feature was rescaled according to predefined minimum and maximum limits before being passed to the learning model.

To further improve model training performance, feature scaling in the form of standardization was applied to the extracted numerical intensity descriptors. By centering the values around the mean and dividing by the standard deviation, all features were brought to a comparable numerical range, which improved convergence speed and prevented dominance of larger-valued variables during training. These statistical operations were carried out in Flutter using list computations and functions available from dart:math. Although the primary decision-making relied on ROI intensity features, image resizing was performed whenever required so that all samples maintained consistent dimensions during storage and processing. This avoided shape mismatches across the dataset and simplified downstream computation. The resizing operation was integrated through the ***img.copyResize()*** function of the Flutter image package. In addition, while the controlled imaging chamber significantly reduced environmental disturbances, mild denoising was occasionally applied to suppress random fluctuations. For this purpose, smoothing filters such as ***img.gaussianBlur()*** and kernel-based enhancement through ***img.convolution()*** were selectively used depending on image quality.

The above sequence of operations was repeated for every captured image across all tested concentrations, resulting in a robust dataset suitable for supervised machine learning. Multiple regression models were evaluated using the processed intensity features, and among them, Polynomial Regression (Degree 3) demonstrated the highest prediction accuracy. This model was subsequently embedded into the Flutter application either through direct Dart implementation of the regression equation or by deployment using the tflite_flutter package for efficient on-device inference. Finally, unknown samples were analyzed using the same portable platform. The smartphone captured the chemiluminescent image, automatically executed the complete preprocessing pipeline, extracted the signal intensity, and instantly estimated the corresponding choline concentration through the trained model.

To improve achievability and data management, the system takes the form of a server-based system, comprising two components: a large language model (LLM) and a local database. The LLM enables high-level query processing and the interpretation of contextual data, allowing the user application interface to be flexible with the analytical backend. Processed data, such as estimated concentrations and signal images, are stored securely to facilitate further analysis and model improvement. This is how the smart chemiluminescent imaging CL system can be used to quantify in real time, automatically, and with a high level of precision, thus avoiding the use of sophisticated laboratory equipments. It consists of a low-cost paper-based sensor with smartphone imaging and machine-learning analytics, an effective field-deployable PoCT solution. This architecture highlights the reality of AI-aided biosensing systems to measure nutritional and food quality, especially in resource-limited settings.

## Results and discussion

This section discussed the optimization of the various chemical parameter required to generate the CL signal for detection of choline. The next part of this section discussed the experimental method for determination of choline with proposed PLC-CL biosensor with various concentration of choline. Further, Stability, reproducibility, and selectivity for the proposed PLC-CL biosensor were investigated and discussed.

### Optimization for CL biosensor

**Fig. 4:**
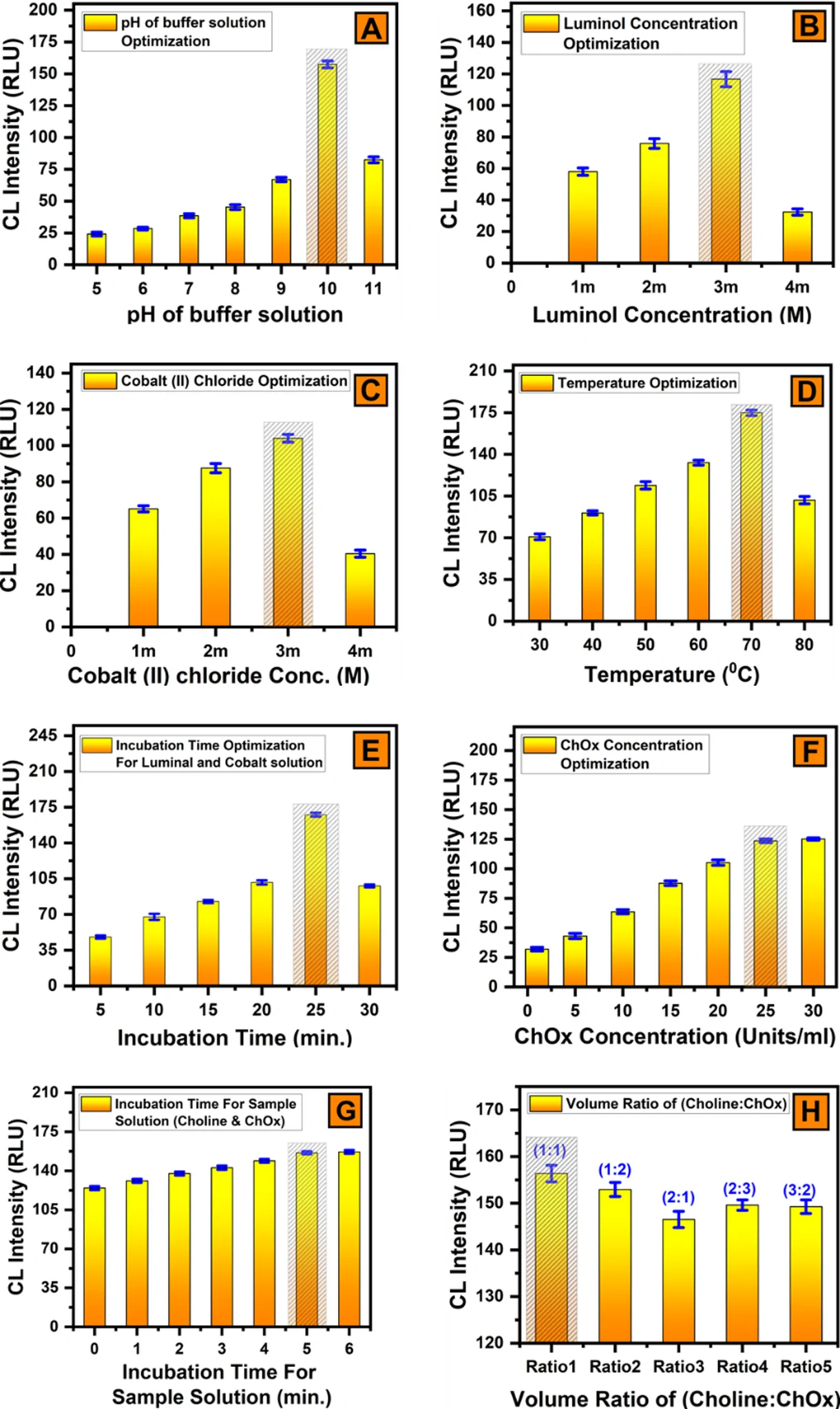
Optimization of key parameters influencing the CL response of the PLC-CL biosensor. (**A**) Effect of pH on CL intensity using Sodium carbonate-Bicarbonate buffer solution as a substrate solution containing luminol and Cobalt(II) Chloride, showing maximum response at pH 10. (**B**) Optimization of luminol concentration, with 3 mM identified as the optimal level. (**C**) Concentration assessment in Cobalt(II) chloride, where 3 mM produced the highest CL signal. (**D**) Optimization of the drying temperature ranged from 30 to 80 °C, revealing 70 °C as the optimal condition for luminol (3 mM), Cobalt(II) chloride (3 mM), and H_2_O_2_ (1 mM). (**E**) Determination of the optimal incubation time for drying the reagent, with 25 min at 70 °C as the maximum. (**F**) Optimization of incubation time of the sample solution (Choline and ChOx) before application on the surface of the sensor. (**G**) The incubation time of the choline/ChOx sample solution was optimized before application of the sensor. Error bars represent the standard deviations of three independent experiments conducted using separate instruments (n = 3) to ensure reproducibility and accuracy. (**H**) Different combinations of Choline: ChOx ratios (1:1, 1:2, 2:1, 2:3, and 3:2) were evaluated while maintaining constant concentrations of choline (1 mM), ChOx (25 units/mL), cobalt chloride (3 mM), and luminol (3 mM) throughout the experiment.

The physicochemical characteristics of the test solution and sample should be optimized well to determine the reproducibility and the stability of the PLC-CL biosensor. The important variables to be considered and optimized would be the pH of a Sodium carbonate-bicarbonate buffer solution, the level of luminol and Cobalt(II) chloride, temperature of the reaction, the time to dry the substrate and incubation time of a sample (choline and ChOx) before detection. It is known that luminol-cobalt and H_2_O_2_ chemiluminescent systems are effective in the alkaline conditions only^45–47^. The pH effect should, therefore, be taken into consideration in order to optimize signal output and repeatability. In this research, Sodium carbonate-bicarbonate buffer solution was used at pH 10 to make the luminol-based substrate but the choline and ChOx samples were pre-treated in PBS at 7.4 pH. The solutions of substrates (Luminol-Cobalt) in the pH range of 5–11 were assessed to identify the pH effect on CL emission. The CL peak was found at pH 10, three different devices (n = 3) were tested and confirmed it as shown in Fig. 4A. The results reveal that the optimum luminol-cobalt-H_2_O_2_ generation is under high alkalinity environment which in turn increases the amount of photons produced. Therefore, it was decided to use Sodium carbonate-bicarbonate buffer solution with pH 10 in preparation of the substrate in further experiments, and maintained the pH level at 7.4 in PBS in order to keep the activity of the enzyme in the choline and ChOx solutions. Measured pH calibration allows optimum chemiluminescent signalling that increases the strength of signal and quality of analysis.

The overall signal intensity and sensitivity of CoCl_2_ depends on the concentrations of luminol and Cobalt(II) chloride—both of which are the basic luminescent reagents in the CL system. As Fig. 4B shows, three devices were used to initially measure the concentration of luminol between 1 and 4 mM (n = 3). The CL intensity increased steadily with increasing luminol concentration within the 1–3 mM concentration range, which is reflective of increased luminescence efficiency and increased electron-transfer kinetics at these two concentration levels. This increase is owed to the increased availability of luminol molecules which are involved in the oxidation reaction producing excited-state intermediates that emit light. On the other hand, the CL signal was greatly reduced in case of the luminol concentration more than 3 mM. This is mainly as a result of a self quenching effect, in which the surplus luminol molecules are involved in non productive reactions, absorbing the excitation energy by non-radiative processes instead of releasing it as photons. This self-quenching behaviour has been reported in previous studies^48,49^. Thus, the concentration of luminol was considered to be 3 mM, as this was the most appropriate concentration between the intensity of the signal and its stability.

A similar optimization experiment was carried out where the concentration of Cobalt(II) chloride was varied whilst keeping the concentration of luminol at the optimized concentration previously. As shown in Fig. 4C, Cobalt(II) chloride has its concentrations systematically increased to 1 mM, 2 mM, 3 mM, and 4 mM to ascertain its impacts on CL intensity. The results show that the increase of the concentration of cobalt (1–3 mM) did not cause dramatic changes to the level of CL emission, which was explained by the catalytic activity of Co^2+^ ions in the decomposition of H_2_O_2_ followed by the increase in reactive oxygen species that catalyze light production and luminol oxidation. Nonetheless, the concentrations that were higher than 3 mM had a little decrease in luminescence probably because of saturation in the catalytic site and an inhibitory effect in breaking the reaction balance^44^. Thus, it was established that both luminol and Cobalt(II) chloride were optimum at 3 mM, which was supported by three replicate devices (n = 3). These modified reagent concentrations produce high-intensity reproducible CL signals, which give a formidable basis of correct, sensitive, and reliable biosensing and analytical detection.

In addition, the Luminol and Cobalt(II) chloride functionalized PLC-CL biosensor was studied in terms of its temperature and incubation time dependency. These factors, when properly controlled, enable the reagents to be stable, the reaction kinetics to be efficient, and the generation of signals to be consistent. To assess the effect of temperature on incubation time, the prepared PLC-CL biosensor was incubated at various temperatures (30–80 °C) at a constant rate of 10 min on three independent PLC-CL biosensor devices (n = 3). Figure 4D shows that the CL intensity increases gradually with the rise in drying temperature and peaks at 70 °C. This optimisation may be explained by the high efficiency of removing excess solvent, the immobilisation of luminol ions and cobalt ions on the paper substrate, which allows for enhanced luminescent activity. Above 70 °C, however, there is a significant reduction in CL intensity which can probably be explained by either partial degradation of temperature sensitive component or changes in paper microstructure that hinder successful interaction of reagent and light emission. Thus, the 70 °C was determined as the best drying temperature and used in further optimisation experiments.

With optimal drying temperature of 70 °C, the incubation time effect was studied. To identify the maximum incubation time that produced the best CL signal, six incubation times, about five to thirty minutes, were taken into consideration. The chemiluminescent response was measured after adding the luminol and Cobalt(II) chloride solutions to the detection area. Figure 4E summarises the results, showing a gradual increase in CL intensity as the incubation time extended, with the highest observation at 25 min. The amplified signal in this range suggests that, under appropriate incubation conditions, we could envision maximum diffusion and contact of the reagents within the porous matrix of the paper. Nevertheless, any incubation after 25 min led to a decline in CL intensity, most likely due to the evaporation of reagents or the possibility of degradation of the substrate and functional layers, which are detrimental to light generation efficacy. Consequently, an incubation period of 25 min at a drying temperature of 70 °C was found to have the most desirable analytical performance. These conditions yielded the most stabilized and maximum CL signal in three repeated devices (n = 3) to ensure an increased sensitivity, reproducibility and reliability of the H_2_O_2_ within the reaction area of the CL biosensor system of the PLC-CL.

The other experimental factors which affect the intensity of the chemiluminescense (CL) signal generated by the biosensor are the incubation period of the choline-ChOx mixture, the concentration of ChOx enzyme, and the specific volumes of the choline and ChOx solutions which should be deposited onto the substrate prior to the reaction. The parameters that were vital were assessed and optimized to calculate the consistency and accuracy of the biosensor outputs using a set of controlled experiments (n = 3). The initial stage was aimed at determining the best concentration of ChOx, the key enzyme to catalyze choline to betaine, which is fundamental in triggering the chemiluminescent cascade. Six different concentrations of enzymes were made between 1 unit/mL to 30 units/mL. In this phase, the concentration of choline was maintained at 1 mM, and the incubation time was set to zero minutes in order to separate the effect of enzyme concentration on the CL response. Experimental results showed that the intensity of CL signal increased consistently as the level of enzyme concentration increased to a peak of 25 units/mL. Above this point, further improvement was not noticed suggesting that the enzyme was saturated. As a result, 25 units/mL was decided upon as the most appropriate concentration of ChOx that would be used in further experiments.

After optimizing the enzyme concentration, the study was then focused on the prolongation of incubation. This parameter controls the time of enzyme–substrate interaction and hence the production of hydrogen peroxide that eventually affects the intensity of light emission. Choline concentration (10 mM) and oxidase concentration (25 units/mL) were set and the incubation time was changed over seven discrete points, i.e. instantaneous measurement (0 min) until 30 min. The findings showed a significant increase in the CL signal as the incubation time was increased and the optimum response was determined at 5 min. Incubation time longer than 5 min did not give any extra signal amplification, confirming 5 min as the adequate time to achieve the best response under the given circumstances.

Finally, the volumetric ratio of the choline and ChOx solutions were investigated to find out which proportion of the reagents would give the maximum chemiluminescent response. Different ratios were tried such as 1:1, 1:2, 2:1, 2:3 and 3:2 where all other factors remained the same. The highest CL intensity was obtained at the 1:1 ratio indicating that an equal volume of choline and choline oxidase solution is the most effective in the enzymatic reaction. All these systematic corrections will have the biosensor operating under conditions that will enhance its sensitivity and choline detection. Each of the parameters, such as pH, reagent concentrations, temperature, and incubation time, was fine-tuned after having been tested meticulously on three devices that were constructed separately. Such optimal conditions are consistent and reproducible because of the consistency of the data as can be indicated by the error bars. This study, thus, formulates a solid ground of an analytical CL-oriented technique of which is sensitive, reproducible and applicable in other analysis scenarios.

### Choline test on PLC-CL biosensors

**Fig. 5:**
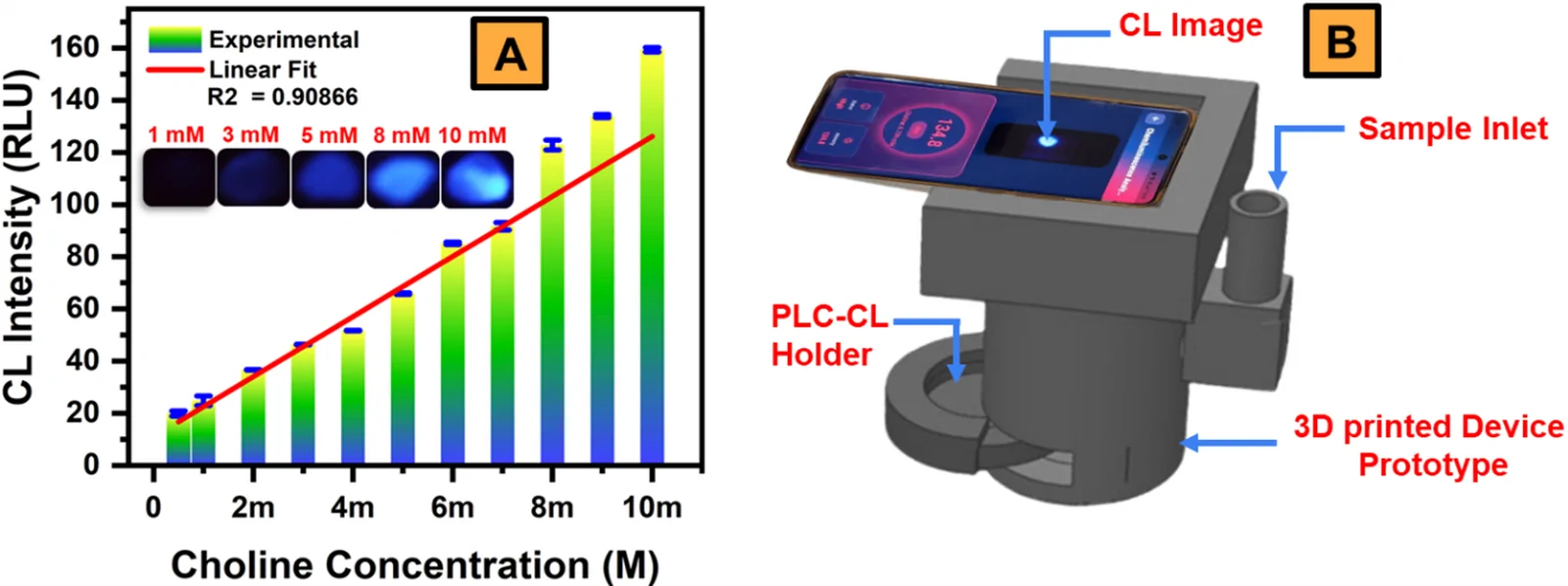
(**A**) Choline detection was performed using the PLC-CL device over a concentration range of 0.5–10 mM, under optimized conditions of Cobalt(II) chloride, luminol, and ChOx. Error bars represent triplicate measurements on separate devices (n = 3). (**B**) A 3D-printed portable CL imaging system integrated with an Android-based application.

The effectiveness of the fabricated PLC-CL biosensor in detecting choline was evaluated through extensive analytical testing conducted in meticulously calibrated experimental settings. The strength of the recorded chemiluminescence signal is consistently and clearly correlated with different choline concentrations, as shown in Fig. 5A. The 3D printed portable CL imaging system integrated with an Android-based application is as illustrated in Fig. 5B). The sensor’s ability to provide accurate quantitative measurements of choline was confirmed by its consistent and proportional increase in light emission with increasing choline concentration. The reported linear detection range of 0.5–10 mM indicates that the biosensor can accurately measure choline over a wide concentration range relevant to practical applications.

**Fig. 6:**
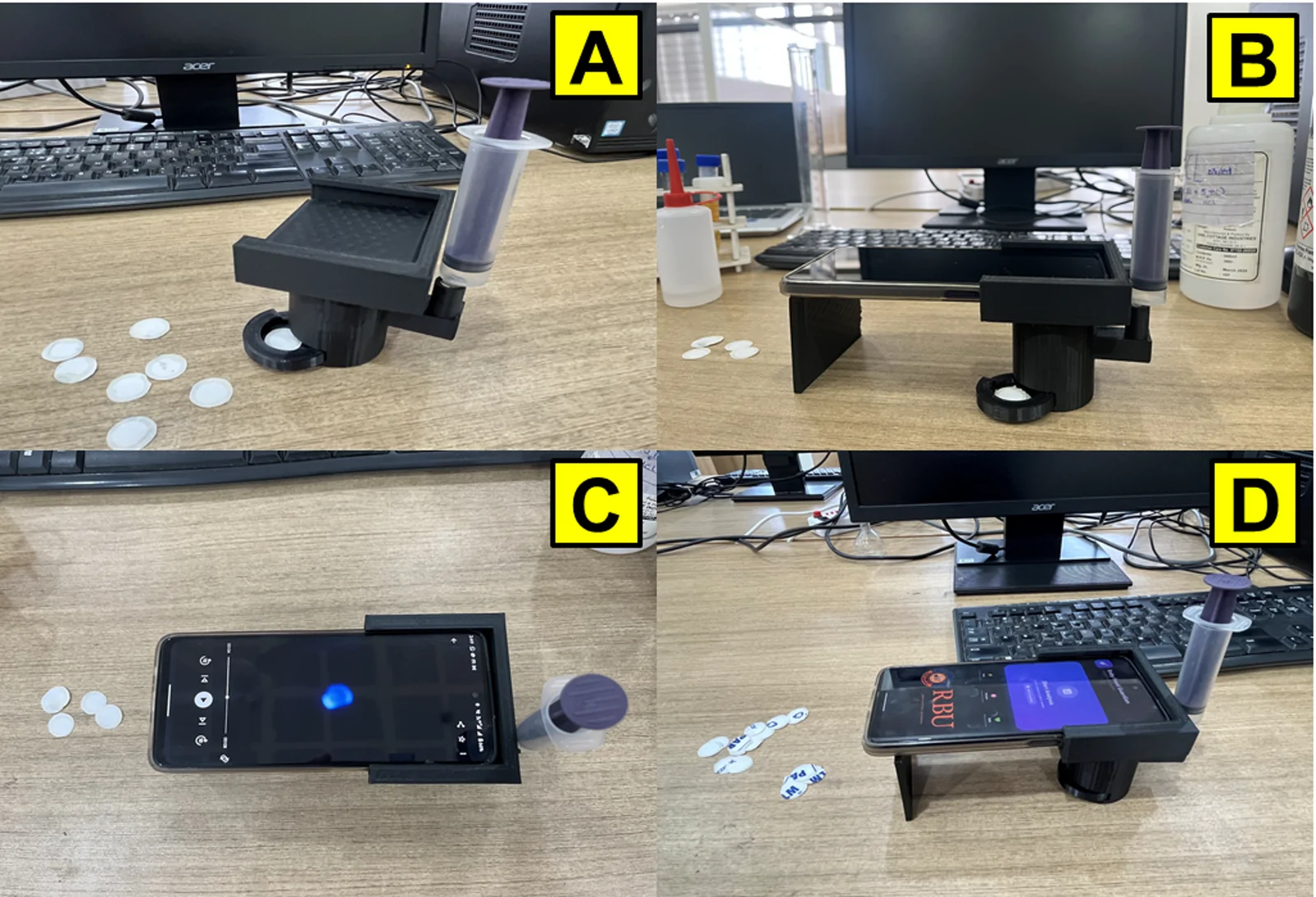
Physical prototype of the smartphone-assisted sensing platform. (**A**) Assembled 3D-printed holder with integrated syringe-based sample loading module and slot for disposable sensing pad. (**B**) Side view of the prototype showing structural support and alignment of the CL sensing region. (**C**) Placement of a smartphone within the holder for signal acquisition, demonstrating illumination and detection interface. (**D**) Fully integrated setup with smartphone in operation, highlighting real-time data acquisition and analysis using a mobile application.

Further, 257.12 µM was calculated as the LOD value of the PLC-CL biosensor. The limit of detection (LOD) was determined based on the standard criterion that the signal must be at least three times greater than the standard deviation of the background noise. The high sensitivity of this sensor design allows detection at very low concentrations, making it possible to reliably identify even smaller amounts of choline. Applications that require the reliable and accurate monitoring of choline concentration, such as quality control of infant food (milk) products and other nutritional checks, benefit from the high degree of accuracy offered by this biosensor. The chemiluminescence process is dependent on the presence of luminol, Cobalt(II) chloride (CoCl_2_), and ChOx as active ingredients in the sensor. In this system, the light intensity is proportional to the amount of Choline as ChOx allows the oxidation of choline to H_2_O_2_. In turn, it is combined with luminol in the presence of Co^2+^ ions. This simple but robust reaction mechanism describes the utility and versatility of the PLC-CL sensor in detecting choline. The Fig. 6 illustrated the actual experimental setup of the smartphone-assisted sensing platform. Figure 6A illustrated an asembled 3D-printed holder with integrated syringe-based sample loading module and slot for disposable sensing pad. The Fig. 6B shows the side view of the prototype showing structural support and alignment of the CL signal sensing region. Placement of a smartphone within the holder for signal acquisition, demonstrating illumination and detection interface as illustrated in Fig. 6C. Figure 6D illustrated fully integrated setup with smartphone in operation, highlighting real-time data acquisition and analysis using a mobile application.

### Repeatability, stability, and interference study with PLC-CL biosensor

**Fig. 7:**
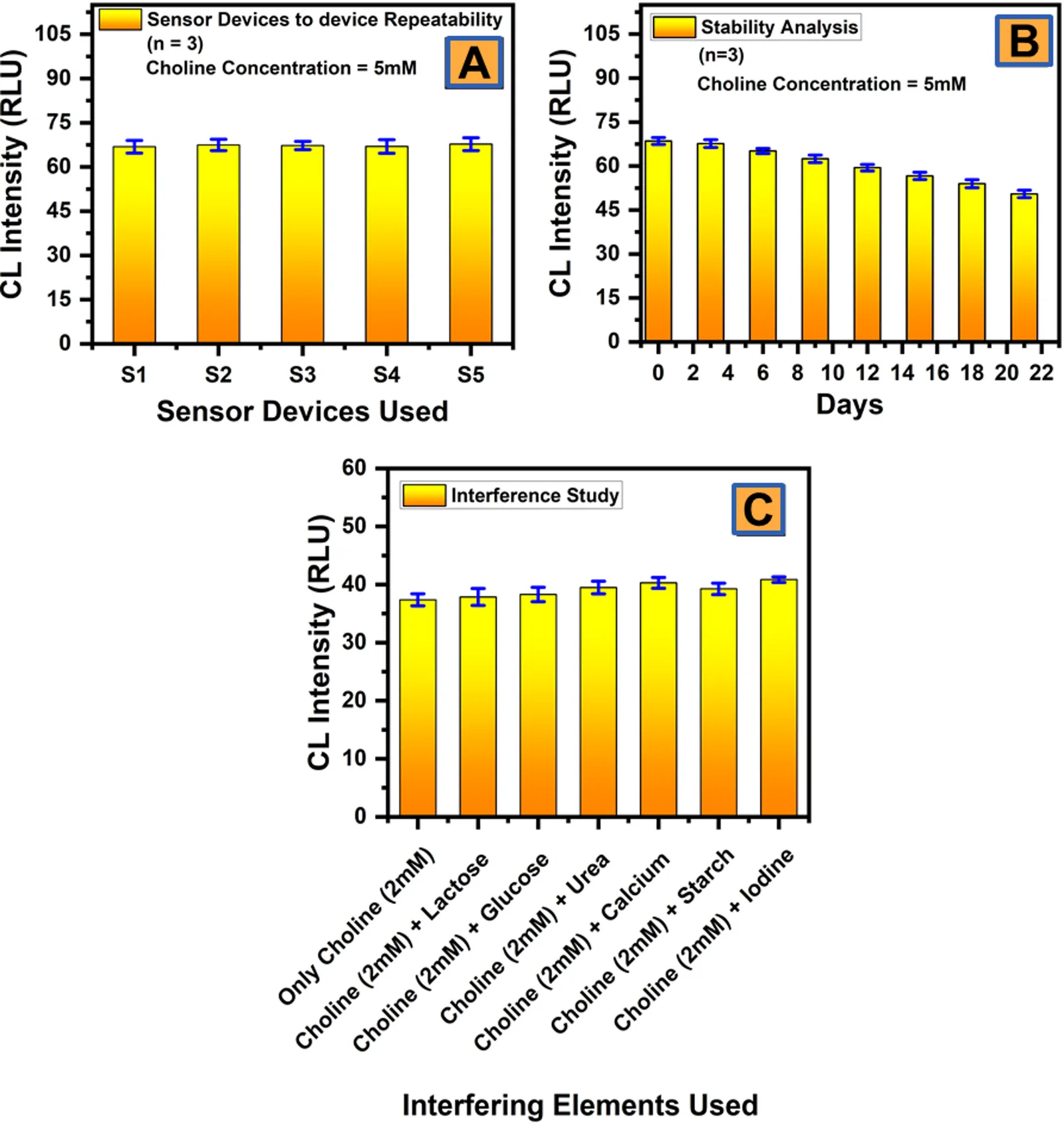
Performance evaluation of the PLC-CL sensor. (**A**) Device-to-device repeatability assessed using choline (5 mM), choline oxidase (25 U/mL), luminol (3 mM), and cobalt chloride (3 mM). (**B**) Stability analysis conducted under identical reagent conditions to assess signal consistency over time. (**C**) Interference study performed with choline (2 mM) in the presence of common interferents—lactose, glucose, urea, starch, iodine, and calcium (500 µM each). Error bars indicate standard deviations from three independent experimental replicates (n = 3).

Stability, reproducibility, and selectivity are important in making sure that investigations conducted using chemiluminescence sensors are reliable. Reproducibility is the capability of a sensor to provide related results when the sensor is subjected to the same conditions. To test this five individual PLC-CL biosensors of three production batches were tested regarding their capacity to detect the presence of choline under the same experimental conditions. The purpose of this evaluation was to assess whether the batches, as differences affecting consistency and sensor analytical reliability in general, may affect the reliability of the results. It was established that the proposed biosensor had good performance across all the devices and therefore makes the proposed choline sensor robust and repeatable in accurate and reliable determination as illustrated in Fig. 7A.

All the devices were assessed by the same procedure discussed above. A 3 mM luminol, 3 mM Cobalt(II) chloride, 25 Units/mL of optimized ChOx enzymes, and 5 mM of fixed concentration of choline were included in this protocol as a reaction system. We carefully tracked and analysed the signals of luminescence generated by each sensor and determined the rate of deviation among different units. The resulting signals of CL reagents were found to be comparable in magnitude under controlled laboratory conditions. This evenness was quantified by calculating the relative standard deviation (RSD) of each sensor to the observed light intensities. The RSD values of the five devices were 3.21%, 2.84%, 2.09%, 3.42%, and 3.22%. This aspect can be best demonstrated by the fact that all of the RSDs were less than the 5 percent mark, which is generally considered a sign of very repeatable sensor performance. The low RSD values shows that the PLC-CL biosensor is reliable, and it can give consistent results even when minute variances in production are present. The control of choline in the real world application requires a sensor that is repeatable across different devices and the sensor should be able to exhibit the same behavior due to its high repeatability.

An important area that must be considered to acertain the viability of a CL biosensor is the stability test that measures the capability of the device to retain its functional capacity within a given time. The research study undertook certain stability tests to determine the long term stability of PLC-CL biosensor in detecting choline. To carry out this experiment, three units of the biosensors were prepared and dried using 3 mM luminol and 3 mM cobalt(II) chloride. The reagents were then heated to 70 °C to ensure sufficient immobilisation and storage stability. The devices were carefully made prior to tests and stored in airtight containers in a room-temperature environment. The functional performance of each biosensor was also monitored by recording the chemiluminescent response at regular intervals (till 21 days). Any abnormalities in signal intensity, which could be symptoms of degeneration and/or activity loss, were monitored by observing the CL signal emitted when the sensors were measured after regular time period interval. The details of stability data are as shown in Fig. 7B. In comparison with the baseline measurement, the biosensors retained more than 90–95% of their signal chemiluminescence during the first six days of testing, demonstrating very good stability. However, after the first week of testing, it became apparent that there was a gradual, though significant, degradation of the signal due to environmental conditions. A very significant decrease of luminescent intensity was observed at the end of 21 days, and this reduction indicates that reagents may wear out or lose activity over time, which affects overall performance. This behavior is primarily attributed to the enzyme-based nature of the sensor and the paper substrate, which are known to be susceptible to gradual loss of activity due to moisture sensitivity and enzyme denaturation. Quantitively, the PLC-CL biosensor to retain more than ~ 90–95% of its initial chemiluminescent response, when stored under specified conditions for a first six days and then decreases the chemiluminescent response (less than ~ 75%) of its initial chemiluminescent response over a two-week period.

High specificity is critical to CL biosensors because, in certain applications, such as infant food testing, different organic compounds can cause interference. The anti-interference capability of the PLC-CL biosensor was critically tested using representative interferents that may commonly occur in milk-based baby foods, including glucose, lactose, calcium, urea, starch, and iodine. These were added at real-life concentrations to determine the possibility of cross-reactivity. The biosensor exhibited no chemiluminescent response when placed in the absence of choline, which validated the inert behaviour of the biosensor with respect to non-target compounds as illustrated in Fig. 7C. This high selectivity indicates that the sensor is well able to differentiate choline from other species sharing the environment, meaning it can detect choline species without interference. This is crucial in ensuring precise detector performance for an analyte in complex, real-world samples.

## Machine learning for PLC-CL sensor validation

ML offers effective means to enhance the accuracy and reliability of sensor validation. ML algorithms can identify intricate patterns, detect anomalies, and predict performance variability that may remain unnoticed by traditional approaches through the analysis of detailed information generated during sensor operation^27,50,51^. A number of ML regression algorithms were applied and tested in this research work as they include Polynomial Regression of degree 3 (PR_Deg3), Support Vector Regression (SVR), Gradient Boosting (GB), Random Forest (RF), Extreme Gradient Boosting (XGBoost), K-Nearest Neighbors (KNN), Decision Tree (DT), and Linear Regression (LR). The overall objective was to achieve the highest prediction accuracy and optimal R^2^ value, ensuring that sensor data interpretation was both strong and precise. Combining machine learning with PLC-CL sensors enables real-time quality control, faster calibration, and defect detection. This method not only optimizes validation procedures but also, ensures uniform sensor performance under varying conditions, making it suitable for sophisticated industrial, environmental, and biological monitoring applications^26,52–54^.

### Dataset statistics

Data augmentation is a practical machine learning approach that involves adding additional information to an existing resource. The training set, from a perspective of increasing the number, albeit not necessarily the variety, without necessarily increasing the amount of data information. It is the process of modifying or transforming existing data samples to create new ones, often by generating multiple examples with varied labels or attributes. For real-world applications such as medical diagnostics and biosensing, where obtaining large, labelled datasets may be challenging, costly, or time-consuming, our method shines. To improve their capacity to generalise to unknown data, machine learning models in supervised learning perform better when trained on large and diverse datasets. It may not be possible to obtain large and balanced datasets in many practical cases, such as biosensor-based diagnostics^55,56^ (e.g., chemiluminescence biosensors). In this case, data augmentation is a crucial tactic for addressing data shortages, enhancing model resilience, and reducing the likelihood of overfitting. The methods used to improve data vary according to the data type. Rotating, flipping, cropping, scaling, adjusting brightness, adding noise, and affine transformations are frequent changes in image-based data^57,58^. In this study, for image augmentation, we have used the maximum degree of rotation as ± 15°, randomly zooming in/out by ± 10%, varying brightness_range from 0.9 to 1.1, setting width_shift_range to 0.05, height_shift_range to 0.05, shear_range to 5, rescaling to 1/255, and flipping horizontally with horizontal_flip = True and vertically with vertical_flip = False. These enhancements can make a machine learning model more resistant to changes in lighting, orientation, or small spatial variations, for instance, when trained on chemiluminescence images obtained by a biosensor incorporated into a smartphone.

### Various machine learning models’ implementation

**Fig. 8:**
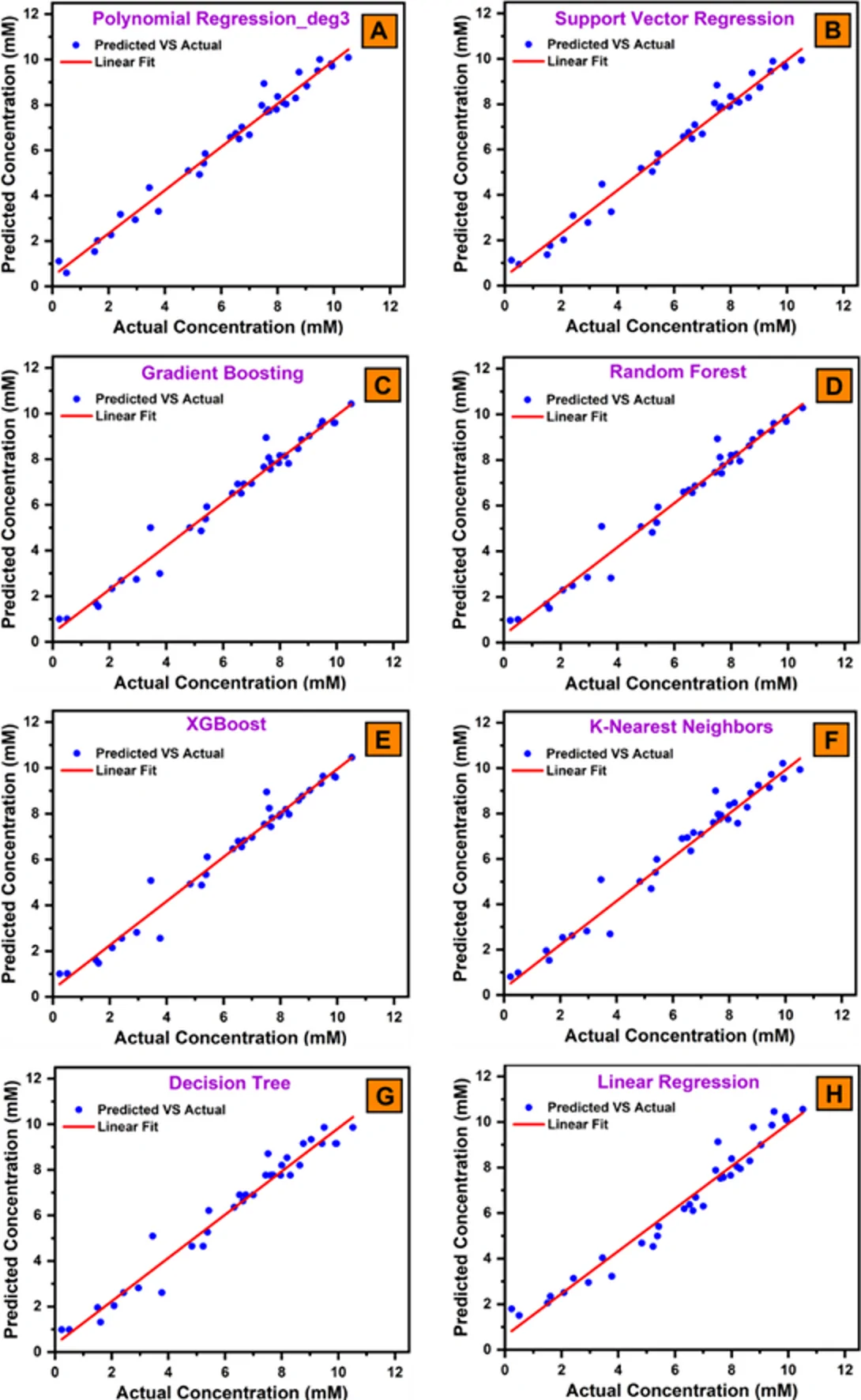
Illustrates the comparative performance of various machine learning algorithms used for predicting choline concentration. The evaluated models include: (**A**) PR_Deg3, (**B**) SVR, (**C**) GB, (**D**) RF, (**E**) XGBoost, (**F**) KNN, (**G**) DT, and (**H**) LR. Each model was trained and tested using the same dataset to assess its predictive accuracy and robustness toward modelling the nonlinear relationship between input features and choline concentration.

Improving the sensor’s accuracy and predicting the choline content were both accomplished through the application of the ML technique to choline detection. The ML models were trained and evaluated using data derived from over 200 trials. While 80% of the datasets were used to train the ML model, 20% were used for testing. The goal of the ML component in this work is not to perform complex image recognition but rather to map extracted intensity features to analyte concentration, which typically requires fewer samples than high-dimensional image classification tasks. With the CL intensity as an input, data from various sensors was used to train the ML models, with a focus on linear data. Figure 8 presents the results of the regression-based analysis conducted. Different machine learning models, including Polynomial Regression of degree 3 (PR_Deg3), Support Vector Regression (SVR), Gradient Boosting (GB), Random Forest (RF), Extreme Gradient Boosting (XGBoost), K-Nearest Neighbors (KNN), Decision Tree (DT), and Linear Regression (LR), were evaluated using metrics like R-squared, MAE, MSE, and RMSE. The following equations are used to obtain the regression metrics^59,60^:

$$Mean\,Absolute\,Error \left( {MAE} \right) = \frac{{\mathop \sum \nolimits_{i = 1}^{N} \left| {Y_{i}^{Predicted} - Y_{i}^{Actual} } \right|}}{N} \ldots \ldots \ldots$$

$$Meaan\,Squared\,Error \left( {MSE} \right) = \frac{1}{N} \mathop \sum \limits_{i = 1}^{N} (Y_{i}^{Predicted} - Y_{i}^{Actual} )^{2} \ldots \ldots \ldots$$

$$Root\,Mean\,Squared\,Error\left( {RMSE} \right) = \sqrt {\frac{{\mathop \sum \nolimits_{i = 1}^{N} (Y_{i}^{Predicted} - Y_{i}^{Actual} )^{2} }}{N}} \ldots \ldots \ldots$$

$$Coefficient\,of\,Determination \left( {R^{2} } \right) = 1 - \frac{{\mathop \sum \nolimits_{i = 1}^{N} (Y_{i}^{Predicted} - Y_{i}^{Actual} )^{2} }}{{\mathop \sum \nolimits_{i = 1}^{N} (Y_{i}^{Predicted} - Y_{i}{\prime} )^{2} }} \ldots \ldots \ldots$$

Table 1 presents the comparative performance metrics of various machine learning regression models developed to predict choline concentration for the proposed affordable, PLC-CL sensor integrated with a smartphone and AI support. The models were evaluated based on standard statistical parameters, including MAE, MSE, RMSE, and R^2^. Among the tested algorithms, RF suggest slight overfitting. GB had a good balance of accuracy and generalisation, with an MAE (Test) of 0.326 and an R^2^ (Test) of 0.975, close to PR, Deg = 3, which had the highest value of R^2^ (Test) at 0.9777, indicating a strong predictive relationship between the experimental variables and choline concentration.

**Table 1:** Presents the comparative performance of various machine learning algorithms used for predicting choline concentration in food samples, supporting the development of an affordable, paper-based chemiluminescence sensor integrated with a smartphone and AI-assisted analytical capabilities.

ML models	Various ML model regression accuracy parameters				
	MAE (train)	MAE (test)	MSE (test)	RMSE (test)	R^2^ score (test)
PR (DEG = 3)	4.366340e−01	0.340510	0.202856	0.450396	0.977739
SVR	4.290845e−01	0.357242	0.208226	0.456318	0.977150
Gradient Boosting	1.668834e−01	0.326437	0.225649	0.475025	0.975238
Random Forest	1.039198e−01	0.319116	0.234697	0.484456	0.974245
XGBoost	8.666347e−02	0.321916	0.272074	0.521607	0.970143
KNN	2.996697e−01	0.433977	0.308876	0.555766	0.966105
Decision Tree	2.331135e−01	0.439175	0.331481	0.575744	0.963624
Linear Regression	5.206662e−01	0.461704	0.391527	0.625722	0.957035

The SVR model was also successful in doing so by having a good balance between training and testing accuracy at a low deviation in MAE and RMSE values. The simpler models, e.g. LR and DT, in turn, had lower predictive power (R^2^ < 0.96) and greater levels of errors that means that they could not represent the linear behaviour of the sensor response. The highest predictive efficiency was found with the models of ensembles and polynomials, which proves the combination of AI with the PLC-CL sensor as an effective approach to detecting choline in food items quickly and without high costs with a smartphone. SVR offers competitive prediction performance but has been slightly outcompeted by a few ensemble based models in respect of error measures and robustness of the model. To incorporate predictive modelling activities in the future regarding the analysis of choline images, we can apply ensemble based models, e.g. Gradient Boosting, since it has the capacity to compute results that have a high level of accuracy because of the complexity of the data that it deals with. Further improvement can be done through optimisation of hyperparameters and advanced feature engineering.

### k-fold cross-validation

In this study, k-fold cross-validation was used to guarantee an effective test of the machine learning models. Data were randomly split into k equal subsets, and training was done repeatedly in which case in one round, one subset was used as the validation, and the rest of the k-1 subsets were used as the training. This was done k times to make sure that all the data points were used in both training and validation. To get a more resistant estimate of both model accuracy and generalization, the performance measures were then averaged over all folds, eliminating reliance on any particular data split.

The Table 2 gives the output of the Polynomial Regression (degree = 3) most effective model tested by k-fold cross-validation that gives the full picture of the predictive ability and consistency of the model. The training MAE is fairly low and stable (mean = 0.4159 ± 0.0119), across the five folds, which is to be expected, meaning that the model does not vary with a wide margin in the training data. The test MAE (0.4273 ± 0. 0507) is a little bit greater than the training MAE which is anticipated and indicates that there is a minimal gap in generalization and no serious overfitting. In further confirmation, the test MSE (0.2659 ± 0.0425) and RMSE (0.5139 ± 0.0417) indicate the moderate value of prediction error with reasonable internal reliability of folds. Despite some differences that can be noted between folds (e.g., Fold 2 and Fold 5 with somewhat more errors), the SDs are small which indicates that the model behaves stably in different data splits. Notably, the R^2^ value in all the folds is very good, the mean being 0.9639 ± 0.0084. This means that the model predicts the information with a high degree of success as it explains more than 96 percent of the differences. There is also a low standard deviation in the R^2^ and this also reflects the strength of the model.

**Table 2:** Polynomial regression (degree = 3) model cross-validation results in fivefold cross-validation.

ML models PR (DEG = 3)	k-fold cross-validation parameters				
	MAE (train)	MAE (test)	MSE (test)	RMSE (test)	R2 score (test)
Fold 1	0.4366	0.3405	0.2029	0.4504	0.9777
Fold 2	0.4103	0.4550	0.2956	0.5436	0.9576
Fold 3	0.4084	0.4496	0.2682	0.5179	0.9658
Fold 4	0.4210	0.4046	0.2382	0.4881	0.9530
Fold 5	0.4031	0.4869	0.3244	0.5696	0.9654
(mean ± std)	0.4159 ± 0.0119	0.4273 ± 0.0507	0.2659 ± 0.0425	0.5139 ± 0.0417	0.9639 ± 0.0084

The results of training and testing errors (MAE, MSE, RMSE) and R2 scores per fold and the mean and standard deviation values, show the accuracy, consistency and ability of the model to generalize to choline quantification.

**Fig. 9:**
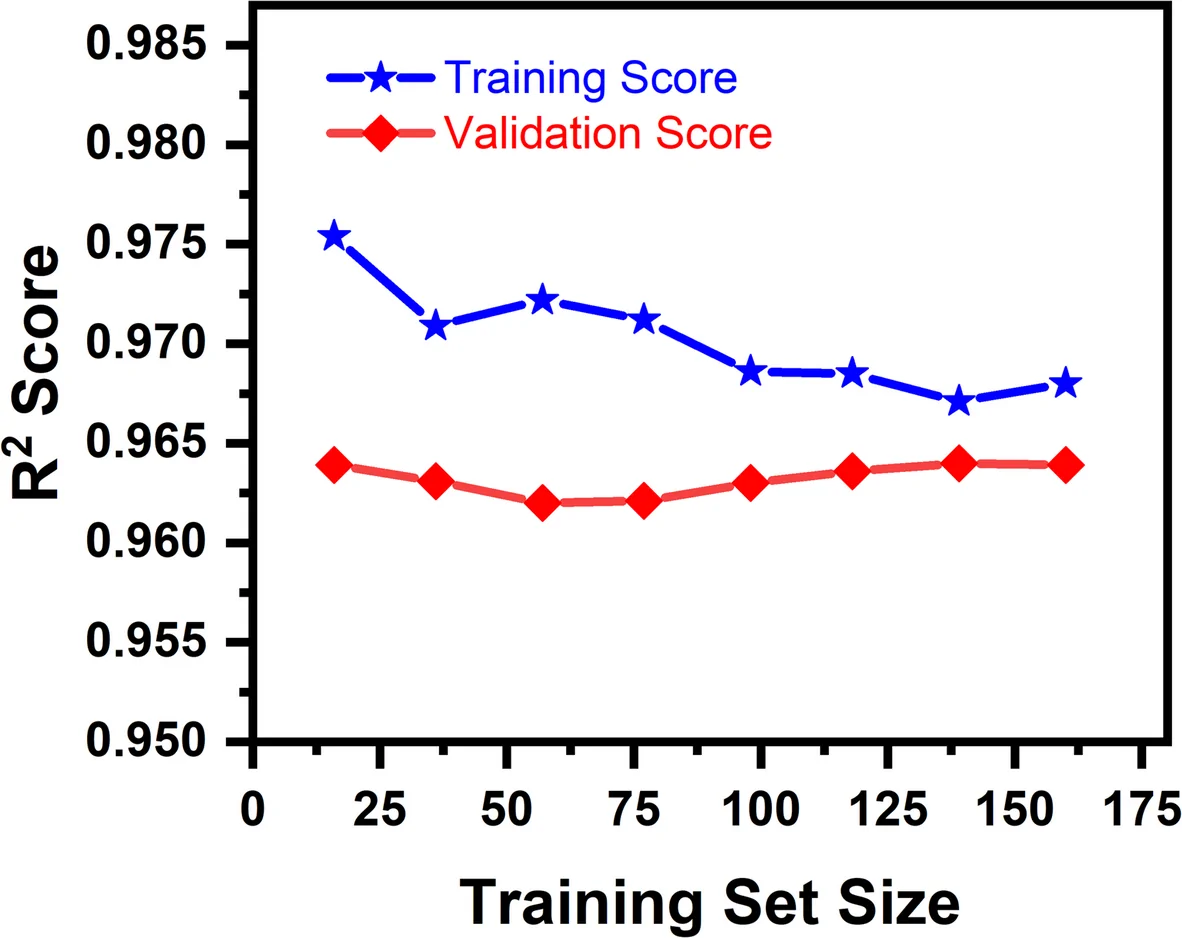
Learning curve analysis of the machine learning model showing training and validation performance as a function of training dataset size. The convergence of training and validation curves with increasing sample size indicates stable model generalization and minimal overfitting. Error bars represent the standard deviation obtained from k-fold cross-validation, demonstrating the consistency and reliability of the model performance.

A learning curve analysis has been conducted to illustrate that performance of the model becomes stable as the amount of training samples tends to the existing size of the dataset as shown in Fig. 9. This implies that the dataset provided is adequate to describe the relationship that exists between chemiluminescence intensity, and choline concentration. The Polynomial Regression (degree 3) model learning curve displays the change in the training and validation R^2^ scores as the size of its training set is increased. The training score (~ 0.975) is relatively high, whereas the smaller (by a small percentage) validation score (~ 0.964) is present, which means that overfitting with a small dataset is present to a small extent. The training score keeps declining, whereas the validation score does not vary too much, although it increases a bit. Notably, the cue of training versus validation scores is also small across, implying that the model is still well-capable of good generalization. Although further samples beyond the 100 samples stabilize, the scores on the training are in the range of bigger numbers, that is, in the range of 0.968–0.969, whereas the validation scores are in the range of 0.963–0.964. This convergence means that there is no overfitting or underfitting of the model.

The relatively narrow regions (standard deviation) further confirm the consistency of model performance across different folds. The learning curve demonstrates that the model achieves stable performance with increasing data and that additional data is unlikely to result in substantial performance gains. The results demonstrate that the Polynomial Regression model provides accurate and consistent predictions with good generalization capability, making it suitable for quantifying choline concentration from chemiluminescent image-derived features.

### Analysis and validation of choline in real milk sample

To assess the performance of the developed PLC-CL sensor on a practical basis, we tested its ability to determine choline levels in fresh milk spiked with choline. We prepared three levels of choline and placed them in milk samples; all these levels fell within the linear range of detection set by the sensor (for n = 3). The measurement of accuracy and precision of the device was verified by using reference concentrations of such spiked samples as reference. Each sample was analyzed using the fabricated PLC-CL. The chemiluminescent images were recorded at every of step the spiked concentrations during the testing process and the intensity of the signal emitted logged. The obtained image was subsequently passed to the ML model to identify the amount of choline concentration in the sample. The results show that the PLC-CL sensor can measure the choline that has been added to fresh milk within a large concentration range. With the 1.5 mM spike in Table 3, the values obtained provide an average of 1.49 mM and percent recovery between 92.53 and 104.93 with a standard deviation (SD) of 0.079 mM and relative standard deviation (RSD) of 5.25. The recovered choline of 3.5 mM was found to be between 103.31 and 111.49 with a standard deviation of 0.120 mM and an RSD of 3.20 meaning that slightly higher reproducibility was achieved. The recoveries of the supreme spike of 7.5 mM were 103.77–111.13 with SD 0.242 mM and RSD 2.99 that was acceptable within the analytical limits. The PLC-CL sensor concentrations were closely compared with the known reference values, which shows very high accuracy and reproducibility of the sensor. This high degree of correlation is used to test how the device is able to detect and quantify choline in a complex media, which in this case is milk, that may have interfering substances. Such results prove the assertion that the sensor is applicable to the real world showing that it can be practiced in the everyday study of choline in food samples and other quality control or nutrition-minded types of monitoring where the absolute analysis of choline is the most important feature.

**Table 3:** Validation of the Choline sample spiked in a fresh milk sample and prediction through Support Vector Machines.

Real sample	Choline detected with the proposed sensor	Percentage recovery	Standard deviation	RSD	Choline detected with ML algorithm
(Fresh Milk + 1.5 mM Choline Spiked)					
S1	1.388	92.53%	0.079	5.25%	1.427
S2	1.524	101.6%			1.601
S3	1.574	104.93%			1.664
(Fresh Milk + 3.5 mM Choline Spiked)					
S1	3.616	103.31%	0.120	3.20%	3.932
S2	3.703	105.8%			4.017
S3	3.902	111.49%			4.208
(Fresh Milk + 7.5 mM Choline Spiked)					
S1	8.25	110%	0.242	2.99%	7.488
S2	8.335	111.13%			7.541
S3	7.783	103.77%			7.251

The Table 4 represents the previously stated choline sensing platforms and focuses on the clear distinctions between the existing PLC-CL sensor that incorporates ML. Classical colorimetric, fluorescence, and electro-chemiluminescent methods can usually measure choline in the nanomolar to low micromolar range with limits of detection, LoD, of 0.0005–0.4 µM, and usually without ML help can be used on milk or serum. In comparison, this system uses a luminol/Cobalt (II) chloride CL system with choline oxidase, and has a linear detection range of 0.5–10 mM and LoD of 257.12 µM/approximately, making it much more suitable to samples with relatively high choline content, like milk. The combination of machine learning facilitates automatic signal analysis and improved validation which had not been documented in the previous works. Despite having a large LoD, particularly relative to other microscale assays, the wide linear range, chemiluminescent readout, and the use of the ML to process the data makes it an excellent choice in practice when it comes to routine quality control and high throughput screening of microfood products such as milk.

**Table 4:** Comparative evaluation of various assays for the detection of Choline.

Bioassay	Linear range	Limit of Detection (LoD)	Operational stability	Selectivity	Matrix	ML used	References
MoS_2_ nanosheets/ChOx	1–180 µM	0.4 µM	Very good chemical stability till 4^th^ day	No interference from 20 mM of uric acid, cysteine, ascorbic acid, urea, cholesterol, glutathione (GSH)	Milk and serum	No	^61^
AChE–ChOx/c-MWCNT/ZrO2NPs/GCE	0.05–200 µM	0.01 µM	Drop of 50% after 60 days	No interference from 1 mM AA, UA, lactic acid, dopamine, heparin	Human Serum	No	^62^
ChOx/GA/TNTs/CH/GCE	0.1–500 µM	0.01 µM	Gradually decreased to 85% of its initial value within 15 days	–	Milk	No	^63^
ChOx/C_60_-PAMAM-PFO/GCE	0.00125–945 µM	0.0005 µM	Decreased 6.8% ECL response after 20 days	No interferences from 20 fold concentration of cholesterol, maltose, lactose, glucose, tryptophan or ascorbic acid	–	No	^64^
L012@SiNPs-NH2	1–5000 μM	1 μM	Good consistency of the ECL signal over 10 days	No interference from dopamine (DA), L-ascorbic acid (VitC), arginine (Arg), glutathione (GSH), glycine (Gly) and cysteine (Cys)	Milk Powder	No	^25^
ZnONR/ChOx, HRP	6–2000 µM	0.0005 mM	Decreased to 78% after 30 days	–	Milk	No	^65^
ChOx/PPyox-PoAP/Pt	Linear up to 100 µM	1 μM	Drop of less than 20%after 40 days	No interference from 0.5 mM UA, 0.2 mM AC, 0.1 mM AA and 0.1 mM cysteine	Human serum and dialysate	No	^66^
ChO/ZnO/Chit/GCE	0.3–5.1 mM	82.5 µM	Significant decrease after 11 days	less than 8% for ascorbic acid, uric acid and citric acid	Breast cancer cells	No	^67^
PANI-UCNPs/ChOx	1–200 µM	0.5 µM	–	No interference from human serum albumin, alkaline phosphatase, Na^+^, K^+^, Ca^2+^ and Mg^2+^	Serum	No	^68^
ChOx/Ni-PB/BG/GCE	4.5 × 10^–7^ to 1.0 × 10^–4^ M	4.5 × 10^−7^ M	Drop of 10% after 30 days	No interference from 0.1 mM AA, UA, glucose, glycine and AC		No	
Luminol/Cobalt(II) Chloride/ChOx	0.5-10 mM	257.12 µM	Drop of more than 25% after 21 days	No interference from glucose, lactose, calcium, urea, starch, and iodine	Milk	Yes	This Work

*MoS*_*2*_ Molybdenum Disulfide, *AChE* acetylcholinesterase, *c-MWCNT* carboxylated multiwalled carbon nanotubes, *ZrO*_*2*_*NPs* zirconium oxide nanoparticles, *MOFs* Metal–organic Frameworks, *GA* Glutaraldehyde, *TNTs* titanate nanotubes, *CH* Chitosan, *GCE* Glassy Carbon Electrodes, *PAMAM* Polyamidoamine, *PFO* polyfluorene derivative poly (9,9-dioctylfluorenyl-2,7-diyl), *ZnONR* Zinc Oxide nanorods, *ChOx* Choline Oxidase, *HRP* Horseradish Peroxidase, *ChOx/PPyox-PoAP/Pt* Choline Oxidase/overoxidized poly(pyrrole)/poly(o-aminophenol), *PANI-UCNPs* Polyaniline-upconverting nanoparticles, *PB* Prussian blue, *BG* bucky gels, *AC* acetaminophen.

## Conclusion

In this study, we successfully developed and tested a low-cost, disposable PLC-CL biosensor that utilises a smartphone to measure the amount of choline in baby food. Choline plays a crucial role in brain growth, neurotransmission, cholesterol metabolism, and the maintenance of cell membranes. To promote positive neurological and physical development during the early years, it is essential to ensure that infant food contains the recommended amount of choline. Our biosensor performs optimally within a detection range of 0.5–10 mM, with a limit of detection (LOD) of 257.12 µM. This demonstrates its applicability in real-life situations, particularly in the management of food quality. The primary novelty of the paper lies in an ML analysis of the data regarding chemiluminescence intensity. This enables one to obtain a more specific and reliable quantification of choline than is possible by conventional calibrations. By incorporating AI-aided prediction, the system is not only more sensitive but also more consistent, which prevents the occurrence of differences that might arise due to the surrounding environment or the sample itself. Additionally, the biosensor is particularly useful in locations with scarce resources, where conventional laboratory methods cannot be employed, as it is easy to use, transportable, and affordable. The method we have developed represents a significant step beyond on-site point-of-care testing in the food industry. It directly influences the issue of ensuring food safety, its nutritional value, and adherence to regulations. Its ease of use and scalability, provided by the paper-based design and the possibility to connect it to smartphones, means that it has a chance of being used by many people. Ongoing work is being conducted to further enhance the biosensor by improving its stability, reducing detection limits, and accelerating reactions, thereby enabling the biosensor to operate with a wide range of complex food matrices. Such ongoing work will play a crucial role in enhancing the biosensor’s performance in diverse circumstances and ensuring optimal performance in field conditions.

The current research shows the viability of constructing a chemiluminescence biosensor based on paper and using it with machine learning in order to quantify choline in a short period. The following stages of the future work will be dedicated to increasing the size of the dataset to enhance the model robustness further and use such advanced methods as a deep learning to extract features automatically. Also, hyperparameter optimization and model refinement can improve the accuracy of prediction and generalization. More will be done to incorporate all the reagents, such as buffering agent, into the paper substrate to eliminate pre-treatment steps of sample and make it user-friendly in real life situations. It is also envisioned that after a fully portable smartphone-based analytical system with a special mobile application will be developed to allow real-time, on-site nutritional monitoring.

## Data Availability

Data is included within this manuscript.
